# Changes in health inequalities for patients with diabetes among middle-aged and elderly in China from 2011 to 2015

**DOI:** 10.1186/s12913-020-05609-4

**Published:** 2020-08-05

**Authors:** Guizhen Cao, Zhizhen Cui, Qinghua Ma, Congju Wang, Yong Xu, Hongpeng Sun, Yana Ma

**Affiliations:** 1 Department of Child Health, Jiangsu Key Laboratory of Preventive and Translational Medicine for Geriatric Diseases, School of Public Health, Soochow University, 199 Ren-ai Rd, SuZhou City, Jiangsu Province 215123 P. R. China; 2 The 3rd People’s Hospital of Xiangcheng District, Suzhou, 215134 P. R. China; 3 Centers for Disease Control and Prevention of Suzhou High-tech Zone, Suzhou, 215000 P. R. China

**Keywords:** Diabetes, Concentration index, Decomposition, Income-related inequality

## Abstract

**Background:**

The purpose of this paper is to measure income-related health inequality among middle-aged and elderly patients with diabetes in China from 2011 to 2015 and to investigate factors that might be related to this inequality.

**Methods:**

The data for this study were obtained from the China Health and Retirement Longitudinal Study that was carried out in 2011, 2013 and 2015. In total, 48,519 Chinese middle-aged and elderly population were included (15,457 in 2011, 16,576 in 2013 and 16,486 in 2015). A principal component analysis was performed to measure asset-based economic status. The concentration index was used to measure income-related inequality in patients with diabetes. Additionally, by used generalized linear model, we decomposed the concentration index to identify factors that explained wealth-related inequality in patients with diabetes.

**Results:**

The prevalence of self-reported diabetes among middle-aged and elderly Chinese was 5.61, 7.49 and 8.99% in 2011, 2013 and 2015, respectively. The concentration indices and 95% confidence intervals for diabetes were 0.1359 (0.0525–0.0597), 0.1207 (0.0709–0.0789), 0.1021 (0.0855–0.0942) in 2011, 2013, and 2015, respectively, which are indicative of inequality that favors the rich. The decomposition of the concentration index showed that residence (39.38%), BMI (31.16%), education (7.28%), and region (6.09%) had positive contributions to the measured inequality in diabetes in China in 2015. Age (− 29.93%) had a negative contribution to inequality.

**Conclusion:**

The findings confirm a health inequality in diabetes that favor the rich. Furthermore, the inequality declined from 2011 to 2015. We suggest that policy and intervention strategies should be developed to alleviate this health inequality, such as narrow the gap between urban and rural areas by improving the urban-rural medical insurance scheme, implementing strategies to enhance hygiene health education, control obesity rate.

## Background

Diabetes is a significant public health issue, which adversely influence the lives of millions of individuals global [1]. In China, a recent study found that the prevalence of diabetes in adults was 10.9%, and approximately 114.4 million people currently suffer from diabetes [2]. It is well known that diabetes is a chronic disease that is influenced by multiple factors. Although physiological or genetic factors play important roles in the condition, socioeconomic characteristics can help us understand inequalities in lifestyle-related disorders such diabetes and its management [3].

A number of recent studies have demonstrated that socioeconomic status (SES) is the most decisive factor affecting health [4–6]. A relation exists between prevalence of diabetes and relatively disadvantaged socioeconomic position in developing and developed countries [7]. People are more prone to have diabetes in high-income countries with a disadvantaged socioeconomic position, while the opposite association has been found for the people of low- and middle-income countries [8, 9]. Additional research report that income, education, occupation, exercise, obesity, behavioral habit are major factors influencing diabetes [10–12]. Furthermore, the high prevalence of diabetes is associated with low level of education [13, 14].

Although there is a study that evaluated influence factors and inequality with respect to the prevalence of diabetes [15], there is not enough evidence to confirm trends of income-related inequality and its determinants of diabetes. Therefore, it is necessary to measure income-related health inequality among the middle-aged and elderly diabetic patients in China and to investigate the factors that might be correlated with this inequality.

## Methods

### Data sources

The data of this study is from the China Health and Retirement Longitudinal Study (CHARLS), which is a national panel data set, conducted by China Center for Economic Research of Peking University [16]. The CHARLS collected high-quality data that represent middle-aged and elderly population over 45 years old and their families in China, covers 150 county-level units, 450 village-level units. A multi-stage stratified probability proportional scale sampling method was used to ensure that the samples were representative of the population [17]. These samples are followed up every two years. In this study, the survey population in 2011, 2013 and 2015 were selected as the research objects. After excluded observations with missing values, 48,519 Chinese middle-aged and elderly population were included (15,457 in 2011, 16,576 in 2013 and 16,486 in 2015). Missing data make up a small amount of overall data (*n* = 1104, 6.7%). After excluding missing values, there is no statistical difference in the basic characteristics of the sample, which does not affect the representativeness of the sample.

### Variables

#### Health outcomes

Each new survey respondent was queried, “Have you ever been diagnosed with diabetes by a doctor?” In follow-up interviews, participants were asked, “Our records from your last interview show that you have had/not had diabetes, is this right?” and “have you been diagnosed with diabetes by a doctor since your last interview in the last 2 years?” Participants recorded yes or no responses to all questions. Respondents who answered “yes” to any questions were required to provide medical or hospital records. People who answered“yes” were classified as having diabetes.

#### Other variables

Ten categories of factors, which may be related to the prevalence of diabetes were used in this study [18], including age, gender, marriage, education, income, residence, region, body mass index (BMI), smoke cigarettes and drinking categories. Marriage was divided into two categories: married and single. The definition of married is married couples live with their spouse or cohabitated. Meanwhile, the definition of single is separated, divorced, widowed or never married. About education, according to the respondents’ highest level of education, the respondents were classified to two categories: illiterate and literate. Illiterate means no formal education. Literate means elementary and higher education, or didn’t finish primary school but capable of reading and writing. Residence was divided into urban and rural areas. According to the division method of the national bureau of statistics, region was divided into eastern and central (most developed and less developed) and western (least developed) regions according to the level of economic development and by province. With regard to smoke cigarettes, according to the respondents who used to smoke at least 100 cigarettes in whole lifetime and currently smoked tobacco products or never smoke, was divided into smoker and nonsmoker. About drink categories, the respondents were classified as drinker and nondrinker, based on whether they consumed beer or any other alcoholic beverage in the past 12 months or never drunk alcohol.

#### SES

To measure inequalities in the prevalence of chronic disease among people with different standards of living, data on household assets and housing characteristics were used to construct a proxy index to measure living standards [19]. In this study, durable consumer goods (containing owning an automobile, electric bike, motorcycle, fridge, washing appliance, television, computer, sound system, video camera, camera, air conditioner, mobile phone, furniture, musical instrument, valuable decorative items, jewelry, collectibles, precious metals, or art works), and housing characteristics (including the type of structure of residence, a one-story or multilevel building, and having a toilet, electricity, running water, bathroom facilities, coal gas or natural gas, heating, a source of cooking fuel, a telephone, and an internet connection) were combined into an index of SES to measure household living standards.

Principal component analysis (PCA) was used to measure the SES of households. PCA is a standard factor analysis method used to describe variation in a set of variables as linear combinations of the original variables, in which each continuous linear combination is derived, to explain variation in the original data as much as possible, while being uncorrelated with other linear combinations [20]. To perform PCA on the variables related to SES, qualitative categorical variables were re-coded as binary variables. Then, all the variables and other continuous variables were entered into the model. SES was classified by weighting the first factor of PCA [20]. In the case of PCA, the wealth index *A*_*i*_ for individual *i* is defined as follows:

$$ {A}_i={\sum}_k\left[{f}_k\frac{\left({a}_{ik}-{\overline{a}}_k\right)}{s_k}\right], $$

where is the value of asset for household, is the sample mean, is the sample standard deviation, and are the weights associated with the principal component, is asset variable.

### Statistical analysis

#### Concentration index (CI)

The CI was used to quantify income-related disparity in the self-reported prevalence of diabetes. The scope of CI was − 1 to 1, where 0 represents no income-related inequality. A positive CI means that health inequality is more pronounced among rich people; a negative CI means that health inequality is more pronounced among poor people [21]. The formula for calculating the CI is as follows:

$$ \mathrm{CI}=\frac{2}{\mu}\mathit{\operatorname{cov}}\left(y,r\right) $$

where *y* is whether an individual has diabetes, *μ* represents the mean of the prevalence of diabetes, and *r* represents the fractional rank of income distribution.

#### Generalized linear model (GLM)

GLM with a binomial distribution and an identity link, which was used to decompose the CI to obtain the contribution rate of each influencing factor to diabetes health inequality, as Y is a binary variable. The GLM is an extension of the linear modelling process that allows models to be fitted to data that follow probability distributions other than the normal distribution, such as the binomial distribution [22]. The link function connects the probability distribution of the outcome variable (the random part of the model) to the systematic (explanatory) part of the model. When the outcome variable follows a binomial distribution, link functions commonly used is the probit, giving rise to probit regression.

#### Decomposition of the CI

The CI was decomposed to determine the selected contributors to inequality in diabetes. In this study, we defined *X*_*k*_ as the socioeconomic factor related to diabetes prevalence. Thus, the linear regression analysis model of diabetes prevalence and related factors is as follows [23]:

$$ {Y}_i=\sum k{\beta}_k{X}_{ki}+{\varepsilon}_i $$

where *Y* denotes the prevalence of self-reported diabetes; the *X*_*k*_ are related to socioeconomic factors, and *ε*_*i*_ is the error term.

The CI may consist of contributions of individual factors to diabetes prevalence inequality. Each contribution is the product of the sensitivity of diabetes prevalence related to the factor and the degree of inequality in that factor. The CI decomposition was calculated as follows [24]:

$$ \mathrm{CI}={\sum}_{\mathrm{k}}\left(\frac{\upbeta_{\mathrm{k}}}{{\overline{\mathrm{x}}}_{\mathrm{k}}}\right){\mathrm{CI}}_{\mathrm{k}}+\frac{GCI_{\varepsilon }}{\overline{y}} $$

where $\overline{y}$ is the mean diabetes prevalence; ${\overline{X}}_k$ is the mean of *X*_*k*_; *CI*_*k*_ is the CI for *X*_*k*_; and *GCI*_*ε*_ is the generalized CI for the error term *ε*.

**Fig. 1:**
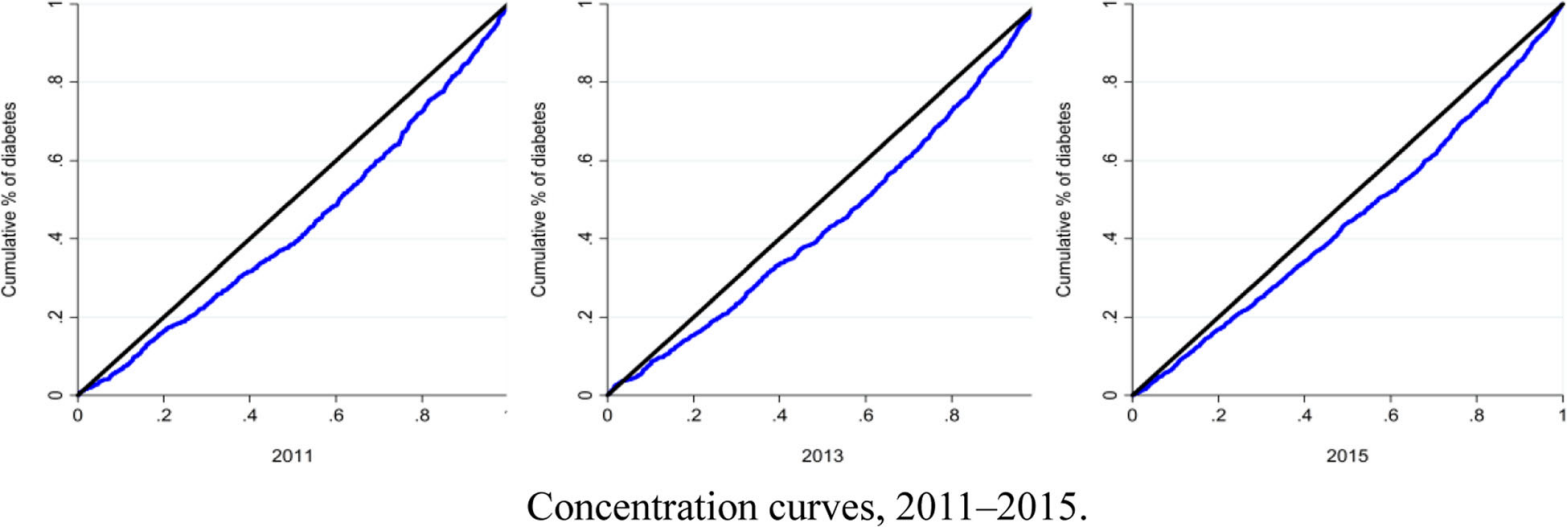
Concentration curves, 2011–2015

All data preparation and analyses were performed in SAS version 9 (SAS Institute Inc., Cary, NC, USA). The CI and the 95% confidence interval were calculated using the bootstrap method. Furthermore, concentration curves in Fig. 1 were obtained using Stata 12.0.

## Results

The demographic characteristics in Table 1 represent the characteristics of the population of middle-aged and elderly population over 45 years old in China. Women comprised slightly over half of the sample in each survey year (Table 1). Per capita household income exhibited no clear trend from 2011 to 2015. The average age increased from approximately 59.5 to 61.6 years, and the proportion of illiterate people and smoker declined during 2011–2015. The prevalence of self-reported diabetes was 5.61, 7.49 and 8.99% in 2011, 2013 and 2015, respectively. Additionally, the CIs and 95% confidence intervals for diabetes were 0.1359 (0.0525–0.0597), 0.1207 (0.0709–0.0789), 0.1021 (0.0855–0.0942), respectively, for the three survey years. These findings were indicative of an inequality that favors the rich and a decreasing inequality from 2011 to 2015. Detailed characteristics of the overall sample are presented in Table 1. Figure 1 shows the concentration curves, which show a general trend that diabetes inequality decreased over time, from 2011 to 2015.

**Table 1:** Detailed description of the patients and concentration indices for diabetes

Variable	2011	2013	2015
	N (%)	N (%)	N (%)
Gender			
male	7429 (48.06)	7869 (47.47)	7792 (47.26)
female	8028 (51.94)	8707 (52.53)	8694 (52.74)
Age (mean ± SD)	59.3 ± 9.6	60.2 ± 9.8	61.6 ± 9.7
Marriage			
married	12,588 (81.44)	13,594 (82.01)	13,308 (80.72)
single	2869 (18.56)	2982 (17.99)	3178 (19.28)
Education			
illiterate	4293 (27.77)	4497 (27.13)	4407 (26.73)
literate	11,164 (72.23)	12,079 (72.87)	12,079 (73.27)
Log-Income (mean ± SD)	4.04 ± 0.67	4.01 ± 0.68	3.85 ± 0.68
Residence			
urban	5839 (37.78)	6266 (37.80)	6160 (37.37)
rural	9618 (62.22)	10,310 (62.20)	10,326 (62.63)
Region			
Central and eastern	10,433 (67.50)	11,083 (66.86)	11,054 (67.05)
West	5024 (32.50)	5493 (33.14)	5432 (32.95)
BMI (mean ± SD)	23.53 ± 3.86	23.81 ± 3.83	23.80 ± 3.75
Smoke			
smoker	6205 (40.14)	5897 (35.58)	4968 (30.13)
non-smoker	9252 (59.86)	10,679 (64.42)	11,518 (69.87)
Drink			
drinker	4808 (31.11)	5336 (32.19)	5271 (31.97)
non-drinker	10,649 (68.89)	11,240 (67.81)	11,215 (68.03)
Diabetes	867 (5.61)	1242 (7.49)	1482 (8.99)
CI	0.1359	0.1207	0.1021
(95% confidence interval)	(0.0525–0.0597)	(0.0709–0.0789)	(0.0855–0.0942)

The regression results are presented in Table 2. The influencing factors of diabetes were age, education, living in urban, and BMI (all *P* < 0.05). The estimated coefficients for the urban variable in 2011, 2013, and 2015 are 0.0229, 0.0312, and 0.0318, respectively, which suggests that urban dwellers are more likely to develop diabetes than their rural counterparts. The estimated coefficients of education for 2011, 2013, and 2015 are − 0.0108, − 0.0131, and − 0.0109, respectively, which suggests that a higher education level is associated with a reduced likelihood of diabetes. The values of the estimated coefficients of urban and BMI suggest that the inequality increased over time. The coefficients of income were 0.0029 and 0.0036 in 2011 and 2013, respectively; however, for 2015, it was − 0.0041.

**Table 2:** The estimated coefficients and *p*-values of explanatory variables for diabetes

Variable	2011		2013		2015	
	Estimate	P-value	Estimate	p-value	Estimate	p-value
Female	0.0016	0.8639	0.0054	0.5536	0.0023	0.7778
Age	0.0015	0.0002	0.0021	0.0001	0.0020	0.0001
Married	0.0036	0.6614	0.0041	0.6350	0.0104	0.1886
Education	−0.0108	0.0162	− 0.0131	0.0092	− 0.0109	0.0146
Income	0.0029	0.5327	0.0036	0.4576	−0.0041	0.3842
Urban	0.0229	0.0013	0.0312	0.0001	0.0318	0.0001
Central and eastern	0.0073	0.2802	0.0102	0.1283	0.0126	0.0539
BMI	0.0063	0.0001	0.0073	0.0001	0.0078	0.0001
Smoker	−0.0036	0.6714	−0.0044	0.6057	−0.0105	0.2018
Drinker	−0.0154	0.0420	−0.0142	0.0771	−0.0168	0.0221

In 2015, the majority of the observed inequalities in the prevalence of self-reported diabetes among middle-aged and elderly adults can be positively attributed to urban (39.38%), BMI (31.16%), education (7.28%), central and eastern region (6.09%), and married (0.95%) (Table 3). Other factors, such as age (− 29.93%) and income (− 4.60%), negatively contributed to inequality. From 2011 to 2015, the impact of BMI on diabetes inequality declined, and the impact of urban, education and income on diabetes health inequality increased. The percent of income in 2011 (3.96%) and 2013 (5.38%) remained positive; however, for 2015 (− 4.60%), it was negative. The implication of this result is that in 2011 and 2013, income is a slightly positive contribution to inequality in diabetes. However, this implication was reduced in 2015. The contribution of income is small and favors the poor, which is largely due to a slight improvement in income inequality.

**Table 3:** The CI of diabetes and the percentage contributions (%) in 2011, 2013, and 2015

Variable	2011		2013		2015	
	CI	Percent (%)	CI	Percent (%)	CI	Percent (%)
Female	0.00743	0.08	0.00385	0.12	−0.00254	− 0.03
Age	−0.01734	−19.88	− 0.02006	−27.57	− 0.02216	− 29.93
Married	0.02492	0.95	0.0277	1.02	0.03784	3.47
Education	−0.21753	8.52	−0.22053	8.64	−0.22953	7.28
Income	0.02576	3.96	0.03339	5.38	0.02673	−4.60
Urban	0.30729	34.83	0.30896	40.31	0.30439	39.38
Central and eastern	0.06181	4.02	0.06093	4.60	0.06613	6.09
BMI	0.01776	4.38	0.01740	33.48	0.01542	31.16
Smoker	−0.04523	0.85	−0.04826	0.83	−0.04071	1.40
Drinker	0.00697	−0.44	0.03601	−1.82	0.04112	−2.41
Residual	−0.03594	32.73	−0.07869	35.01	−0.09459	48.19
Total	0.1359	100	0.1207	100	0.1021	100

Table 4 shows the results of decomposition. The contributions of education and BMI to the change are 10.84 and 38.95% in 2015–2011, respectively, and 9.27 and 26.59% in 2015–2013, respectively. The contributions of income and urban change are 26.39 and 18.61% in 2015–2011, respectively, and 34.64 and 26.13% in 2015–2013, respectively.

**Table 4:** The contributions of variables to the CI and the change from previous years to 2015

Variable	Contribution to CI			Change (%)	
	2011	2013	2015	2015–2011 2015–2013	
Female	0.00011	0.00015	−0.00003	0.36	0.55
Age	−0.02701	−0.03328	− 0.03057	9.32	−8.39
Married	0.00129	0.00123	0.00355	−5.92	−7.18
Education	0.01158	0.01044	0.00744	10.84	9.27
Income	0.00538	0.00649	−0.00470	26.39	34.64
Urban	0.04733	0.04866	0.04022	18.61	26.13
Central and eastern	0.00546	0.00555	0.00622	−1.99	−2.07
BMI	0.04671	0.04042	0.03183	38.95	26.59
Smoker	0.00115	0.00100	0.00143	−0.73	−1.33
Drinker	−0.00059	−0.00220	− 0.00246	4.89	0.80

## Discussion

Our study reveals health inequalities with respect to diabetes in China from 2011 to 2015. The findings confirm that an inequality in diabetes is more pronounced among the rich, and the trend of inequality has declined in the last few years. We identified marked residence and education differences to the extent to which health inequality exists among individuals with different levels of wealth. The decline in health inequalities during 2011–2015 was largely due to education, income and BMI.

Residence is a key factor in explaining health inequality in diabetes. We found that people living in urban have higher prevalence of diabetes, which is consistent with some previous studies [12, 25]. The huge economic difference between urban and rural can explain this finding [26]. Generally speaking, the higher prevalence of diabetes among urban residents can be attributed to their sedentary lifestyle which usually lacks physical labor. Furthermore, compared with urban residents, rural residents are more sensitive to healthcare utilization and the cost of chronic diseases, because of low incomes, lack of medical security mechanisms, and poor healthcare awareness [27]. So, the self-reported prevalence of diabetes is lower in rural areas, as respondents may not know they have diabetes. Therefore, it is necessary to narrow the gap between urban and rural areas by improving the urban-rural medical insurance scheme.

Several studies have suggested that a high level of education is associated with a low prevalence of diabetes [13]. This finding is consistent with the results of this paper. A higher educational level is an indicator of the ability to translate information into practical behaviors and thus to regularly manage and control chronic diseases [9]. One possible reason is that people with higher education may have better health literacy to fight against the risk factors of diabetes, such as harmful diet, excessive drinking and lack of exercise.

In this study, from 2011 to 2015, residence in urban area and education reduced the inequalities of diabetes, and the possible reasons are as follows. First, the process of urbanization is increasing, and a large number of rural people are moving into cities. Second, in recent years, the living standard and the healthcare awareness of farmers are rapidly increasing, while the physical labor intensity is decreasing year by year, resulting in the self-reported prevalence of diabetes in rural areas increasing. Third, with the improvement in education level, people’s awareness of self-health management is strengthened.

In addition, this study also found a contribution of region to inequality. Unbalanced socioeconomic development has caused regional disparity to become an important factor affecting health inequality. This study shows that the prevalence of diabetes among middle-aged and elderly people in the central and eastern regions is higher than in the western regions. First, the aging population in economically developed areas is higher than that in the western regions, and the prevalence of chronic diseases is higher. Second, the level of education and health awareness and the degree of recognition of diseases are higher than those in the western regions, and self-reported diseases are high.

BMI is a significant contributor to inequality in diabetes. Overweight people have a higher risk of developing diabetes than people with a normal BMI. People with high income levels may consume more fat, meat and processed foods and are more likely to be overweight [28]. Therefore, it is necessary to promote a healthy lifestyle among these at-risk individuals.

This study calculates a CI of diabetes inequality, and the CI is decomposed into the contributions of individual factors to diabetes prevalence inequality, in which each contribution is the product of the sensitivity of diabetes prevalence with respect to that factor and the degree of inequality in that factor. Furthermore, this study compared the inequality trend of diabetes from 2011 to 2015 in china. These results can provide some inspiration for the inequality of diabetes and the reasons for it.

Policies for reducing the inequality of diabetes incidence ought to focus on the socioeconomic factors, such as implementing strategies to enhance hygiene health education, promote healthy lifestyles to control obesity rate, narrow the disparity between urban and rural areas [23].

A strength of this study was that the GLM with a binomial distribution and an identity link was used to decompose health inequality. In this study, the linear decomposition method of Wagstaff (2003) was adopted, and the GLMs with binomial distribution and identity link was used to decompose the CI to obtain the contribution rate of each influencing factor. GLM method can generate effective estimates that do not change with the selection of the reference group, so as to obtain the stable contribution rate of each influencing factor to determine the contribution degree of various factors to health inequality.

This study also has several limitations. First, we used a subjective evaluation indicator of self-reported health instead of objective indicators such as clinical examination, the true diabetes prevalence might have been underestimated to some extent. Because of the inadequate understanding of health status in a self-assessment questionnaire, some people with diabetes do not know that they have diabetes. Second, a percentage of our sample of individuals was excluded from the analysis due to missing data. However, the missing data make up a small amount of overall data, participants with missing data were (*n* = 1104, 6.7%). Furthermore, we compared the deleted data with the remaining data and found no significant differences in their characteristics. Therefore, missing data not enough to affect the proportion of missing data in the study was low, and did not affect the objectivity and correctness of the overall results. Third, the data we utilized is based on self-reports of diabetes, no distinction between types of diabetes. However, type 2 diabetes accounts for about 90% of diabetic patients. Therefore, the results of this study are objective. Finally, whether patients with diabetes used anti-diabetic medications was not considered in this study, which might result in some biases.

## Conclusion

In conclusion, the findings confirmed a health inequality in diabetes that favor the rich, and the inequality declined from 2011 to 2015. Furthermore, a substantial portion of the inequality was explained by residence, BMI, and education. Thus, to reduce inequality in diabetes, intervention policies should focus on these factors, such as narrow the gap between urban and rural areas by improving the urban-rural medical insurance scheme, strengthen hygiene health education, promote healthy lifestyles to control obesity rate. In addition, the active collaboration of the health system with other social and economic sectors could be an effective strategic policy for overcoming the barriers of socioeconomic inequalities in diabetes.

## Data Availability

This data was drawn from the data that were derived from the China Health and Retirement Longitudinal Study (CHARLS) conducted from 2011 to 2015. They are opened to everyone. Researchers who want to use these data can visit: http://charls.pku.edu.cn/en.

## Abbreviations

SES: Socioeconomic status
CI: Concentration index
CHARLS: China Health and Retirement Longitudinal Study
BMI: Body mass index
PCA: Principal component analysis
GLM: Generalized linear model
